# MiR-10a in Pancreatic Juice as a Biomarker for Invasive Intraductal Papillary Mucinous Neoplasm by miRNA Sequencing

**DOI:** 10.3390/ijms22063221

**Published:** 2021-03-22

**Authors:** Natsuhiko Kuratomi, Shinichi Takano, Mitsuharu Fukasawa, Shinya Maekawa, Makoto Kadokura, Hiroko Shindo, Ei Takahashi, Sumio Hirose, Yoshimitsu Fukasawa, Satoshi Kawakami, Hiroshi Hayakawa, Hitomi Takada, Natsuko Nakakuki, Ryoh Kato, Tatsuya Yamaguchi, Yasuhiro Nakayama, Hiromichi Kawaida, Hiroshi Kono, Taisuke Inoue, Tetsuo Kondo, Daisuke Ichikawa, Nobuyuki Enomoto

**Affiliations:** 1 First Department of Internal Medicine, Faculty of Medicine, University of Yamanashi, Chuo, Yamanashi 409-3898, Japan; nkuratomi@yamanashi.ac.jp (N.K.); fmitsu@yamanashi.ac.jp (M.F.); maekawa@yamanashi.ac.jp (S.M.); makotok@yamanashi.ac.jp (M.K.); shindoh@yamanashi.ac.jp (H.S.); etakahashi@yamanashi.ac.jp (E.T.); sh99073@yahoo.co.jp (S.H.); ii258pp2@yahoo.co.jp (Y.F.); skawakami@yamanashi.ac.jp (S.K.); minaminoro73@gmail.com (H.H.); thitomi@yamanashi.ac.jp (H.T.); natsukon@yamanashi.ac.jp (N.N.); katohr@yamanashi.ac.jp (R.K.); ytatsuya@yamanashi.ac.jp (T.Y.); ynakayama@yamanashi.ac.jp (Y.N.); tinoue@yamanashi.ac.jp (T.I.); enomoto@yamanashi.ac.jp (N.E.); 2 First Department of Surgery, Faculty of Medicine, University of Yamanashi, Chuo, Yamanashi 409-3898, Japan; kawaidah@yamanashi.ac.jp (H.K.); hkouno@yamanashi.ac.jp (H.K.); dichikawa@yamanashi.ac.jp (D.I.); 3 Department of Pathology, Faculty of Medicine, University of Yamanashi, Chuo, Yamanashi 409-3898, Japan; ktetsuo@yamanashi.ac.jp

**Keywords:** intraductal papillary mucinous neoplasm, miRNA, pancreatic juice, formalin-fixed paraffin-embedded samples, next-generation sequencing

## Abstract

New biomarkers are needed to further stratify the risk of malignancy in intraductal papillary mucinous neoplasm (IPMN). Although microRNAs (miRNAs) are expected to be stable biomarkers, they can vary owing to a lack of definite internal controls. To identify universal biomarkers for invasive IPMN, we performed miRNA sequencing using tumor-normal paired samples. A total of 19 resected tissues and 13 pancreatic juice samples from 32 IPMN patients were analyzed for miRNA expression by next-generation sequencing with a two-step normalization of miRNA sequence data. The miRNAs involved in IPMN associated with invasive carcinoma were identified from this tissue analysis and further verified with the pancreatic juice samples. From the tumor-normal paired tissue analysis of the expression levels of 2792 miRNAs, 20 upregulated and 17 downregulated miRNAs were identified. In IPMN associated with invasive carcinoma (INV), miR-10a-5p and miR-221-3p were upregulated and miR-148a-3p was downregulated when compared with noninvasive IPMN. When these findings were further validated with pancreatic juice samples, miR-10a-5p was found to be elevated in INV (*p* = 0.002). Therefore, three differentially expressed miRNAs were identified in tissues with INV, and the expression of miR-10a-5p was also elevated in pancreatic juice samples with INV. MiR-10a-5p is a promising additional biomarker for invasive IPMN.

## 1. Introduction

Intraductal papillary mucinous neoplasm (IPMN) is a noninvasive mucin-producing epithelial neoplasm, which is grossly visible, predominantly papillary, and rarely flat and which grows in the main or branch pancreatic ducts [1,2]. The prevalence of incidental findings of IPMN or pancreatic cyst increased to 4.3% with improved diagnostic imaging technologies [3,4]. IPMN, like pancreatic intraepithelial neoplasia (PanIN), is a lesion, which is a precursor of pancreatic ductal carcinoma, and shows a wide-ranging histological spectrum, from low-grade dysplasia (LGD) and high-grade dysplasia (HGD) to IPMN with an associated invasive carcinoma (INV) [1,5]. IPMN with INV exhibits a poor survival rate after resection when the tumor is accompanied by lymph node metastases or histological findings of tubular adenocarcinoma [6,7]. The five-year survival rate of INV after resection (30%) is much poorer than that of LGD and HGD (73% and 70%, respectively) [8]. Therefore, diagnosing INV without delay is extremely important.

To provide an optimal management algorithm for IPMN, the International Consensus Guidelines (ICG) for the management of IPMN and mucinous cystic neoplasms of the pancreas were established and revised in 2006 and 2012 [9,10] and most recently in 2017 [11]. The ICG stratify risks for malignant IPMN by mainly using the mural nodule size, pancreatic duct diameter and cyst size, which can also be assessed with regular imaging tests, such as computed tomography, magnetic resonance imaging and endoscopic ultrasonography. The sensitivity of diagnosing malignant IPMN, including HGD and INV, has improved with the revisions of the ICG to more than 90%; however, the specificity remains less than 30%, which can lead to overtreatment in patients with IPMN [12]. Further, 9% of patients with malignant IPMN would be treated as low risk, and their diagnoses would be missed [12,13,14]. Conversely, pancreaticoduodenectomy and total pancreatectomy are relatively invasive treatments with treatment-related mortalities of 0–4.9% and 0–8%, respectively, and they require careful indication [15,16,17]. Additional malignant predictors, including new biomarkers, are required to select an optimal treatment for IPMN.

Recent developments in biological analysis made amplifying target genes and performing a comprehensive gene analysis by DNA and RNA sequencing possible even from unstable and small clinical samples, including pancreatic tissues and pancreatic juice samples [18,19,20]. Compared with the use of other biological molecules such as proteins, DNAs and other RNAs as biomarkers, microRNA (miRNA) demonstrates the advantage of being very stable [21,22]. MiRNAs are small, noncoding RNAs consisting typically of 18–23 nucleotides, which can base-pair with complementary sequences in mRNA to silence RNA and post-transcriptionally regulate gene expression [23], such as during tumorigenesis and metastasis [24]. The molecular functions [25,26] and usefulness of these molecules as potential biomarkers [27,28,29,30] in pancreatic cancer have been reported, which suggests that new miRNA biomarkers for malignant IPMN could be found. However, one disadvantage of miRNAs as biomarkers is their lack of a definite internal control, which leads to inconsistent results across different studies.

In this study, in order to identify miRNA biomarkers for malignant IPMN, we comprehensively analyzed the miRNA expression using next-generation sequencing (NGS) in tumor tissues paired with normal tissues and validated the usefulness of these biomarkers using pancreatic juice samples.

## 2. Results

### 2.1. Quantification of miRNAs Extracted from Clinical Samples

MiRNA sequencing was performed, and an average of 36,485 and 34,168 miRNA reads from the sequence reads of formalin-fixed paraffin-embedded (FFPE) and pancreatic juice, respectively, were mapped to the reference genome (hg19) (Table S1). Of 2792 total miRNAs, we eliminated immature miRNAs and miRNAs with extremely low expression levels (no more than an average of three reads), leaving 194 and 162 miRNAs from tissue and pancreatic juice samples, respectively, for subsequent analysis (Table S1).

### 2.2. Differential miRNA Expression in IPMN by NGS Analysis

**Figure 1:**
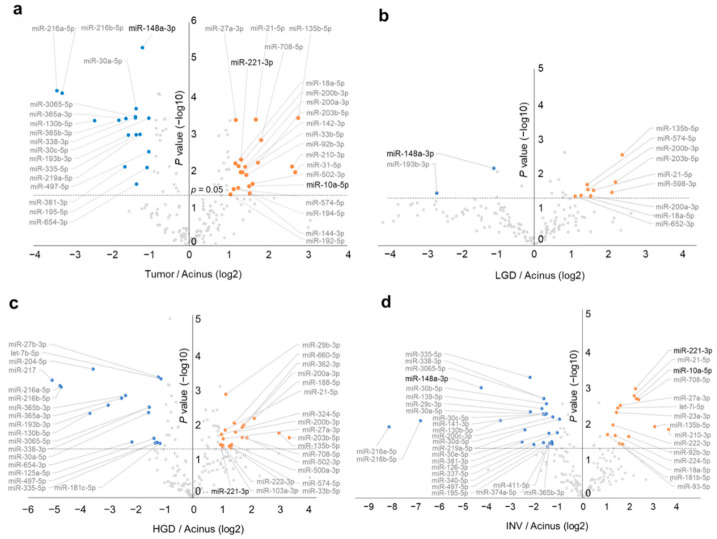
Volcano plots of next-generation sequencing data show differential miRNA expressions in intraductal papillary mucinous neoplasm (IPMN) between tumor tissue and its paired normal acinus tissue. (**a**) All IPMN cases (*n* = 19). (**b**) Cases with low-grade dysplasia (LGD) (*n* = 4). (**c**) Cases with high-grade dysplasia (HGD) (*n* = 9). (**d**) Cases with invasive carcinoma (INV) (*n* = 6). Orange dots show significantly upregulated miRNAs (fold-change > 2, *p* < 0.05), and blue dots show significantly downregulated miRNAs (fold-change < 0.5, *p* < 0.05).

After normalization of miRNA sequence reads by calculating the percentage of reads in the total sample for each miRNA, we further normalized the results for 194 miRNAs by calculating the ratio of the miRNA expression in the tumor tissue to the paired normal acinar tissue in 19 tumor-normal paired tissue samples (two-step normalization). Next, we performed a volcano plot analysis with paired t-tests and fold changes of miRNA expressions, and we revealed 37 miRNAs that were differentially expressed between tumor and normal acinus tissues (20 upregulated and 17 downregulated in tumor tissue) (Figure 1a). Next, we further revealed 11 miRNAs that were differentially expressed in tumor tissues compared with normal acinar tissues in LGD (nine upregulated, two downregulated), 36 in HGD (19 upregulated, 17 downregulated) and 41 in INV (15 upregulated, 26 downregulated) (Figure 1b–d), with a tendency for an increase in the number of differentially expressed miRNAs with an increased histological malignancy. Overall, we identified 69 unique differentially expressed miRNAs.

### 2.3. MiR-10a and miR-221 were Upregulated in Invasive IPMN

**Figure 2:**
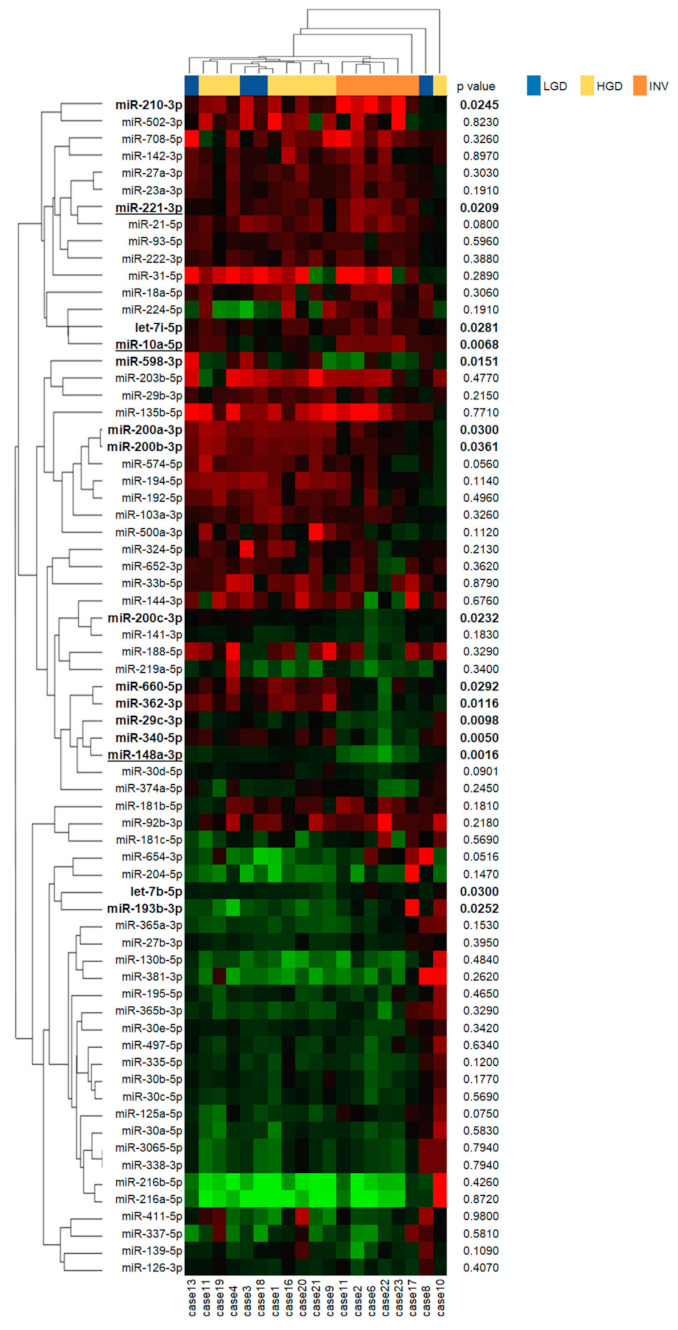
Heatmap of miRNA expressions stratified by pathological grades of intraductal papillary mucinous neoplasm (IPMN). The expressions of each miRNA in the IPMN tissues are shown as a heatmap. The expression levels of miRNAs are shown on a green-red scale in the center panel where the green and red should be interpreted as a low and high expression, respectively. The right panel shows the *p*-values by a Kruskal–Wallis test comparing three pathological grades (bold *p* value < 0.05). The left and upper panels show miRNAs and patients that were arranged according to an unsupervised hierarchical clustering, which was performed using a one minus Pearson’s correlation as the distance measure and an average linkage as the agglomerative method. LGD, low-grade dysplasia; HGD, high-grade dysplasia; INV, invasive carcinoma. MiRNAs with underlines show significant differential expressions between INV and non-INV (LGD and HGD) patients.

**Figure 3:**
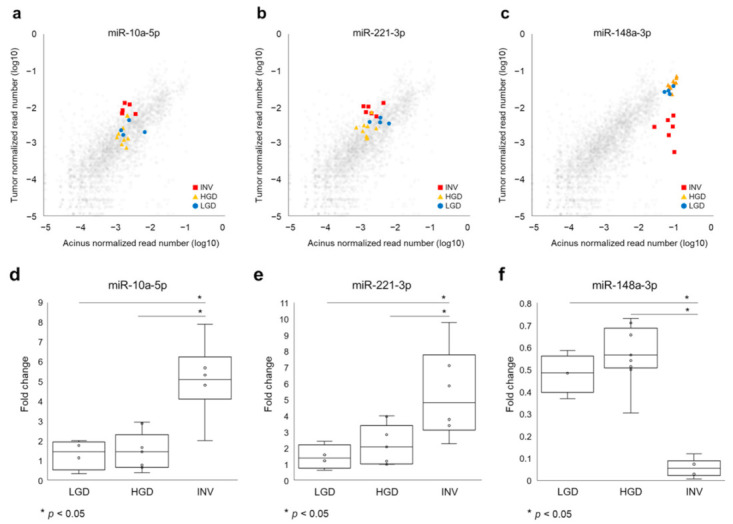
Candidate biomarker miRNAs in intraductal papillary mucinous neoplasm (IPMN) tissues to differentiate invasive IPMNs from noninvasive IPMNs. (**a**,**d**) MiR-10a-5p and (**b**,**e**) miR-221-3p were significantly upregulated in invasive carcinoma (INV) compared to low-grade dysplasia (LGD) and high-grade dysplasia (HGD). (**c**,**f**) MiR-148-3p was downregulated in INV compared to LGD and HGD. Gray dots indicate all other normalized read numbers of miRNA for each grade.

Next, we compared the 69 differentially expressed miRNAs between the three histological grades and showed the difference of miRNA expressions among histological grades as a heatmap with *p*-values (Figure 2). Statistical analyses of the tumor: the normal ratios of the miRNA expression levels among the three grades revealed 15 differentially expressed miRNAs (miR-210-3p, miR-221-3p, let-7i-5p, miR-10a-5p, miR-598-3p, miR-200a-3p, miR-200b-3p, miR-200c-3p, miR-660-5p, miR-362-3p, miR-29c-3p, miR-340-5p, miR-148a-3p, let-7b-5p and miR-193-3p), as shown in Figure 2. Further, we identified that miR-10a-5p (Figure 3a,d) and miR-221-3p (Figure 3b,e) were upregulated in invasive IPMN (INV) when compared with noninvasive IPMN (LGD and HGD) and that miR-148a-3p (Figure 3c,f) was downregulated in noninvasive IPMN by post hoc analyses, which compared each of two groups after a comparison of the three groups (Table 1). No miRNAs were differentially expressed between LGD and HGD.

### 2.4. MiR-10a was Revealed to Be a Malignant Biomarker of IPMN in Pancreatic Juice

**Figure 4:**
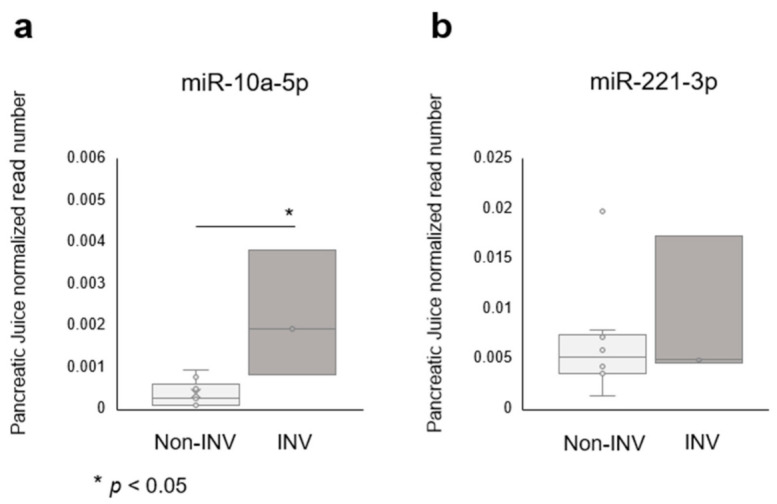
The miRNA expression of candidate biomarkers in intraductal papillary mucinous neoplasm (IPMN) pancreatic juice samples. (**a**) MiR-10a-5p was significantly upregulated in invasive IPMN compared to noninvasive IPMN, and (**b**) miR-221-5p showed a similar tendency. INV, invasive carcinoma.

We validated these identified miRNAs in preoperative pancreatic juice samples, which were taken from near the tumor and had the potential to contain biomarkers for malignant IPMNs. A volcano plot analysis of miRNAs in pancreatic juice samples revealed that five miRNAs (miR-10a-5p, miR-106b-5p, miR-197-3p, miR-664a-3p and let-7d-3p) were upregulated in IPMN associated with INV, compared with LGD and HGD. Of the miRNAs that were elevated in tissue samples, miR-10a-5p was upregulated in IPMN associated with INV when compared with HGD or LGD (*p* = 0.002, fold-change; 7.2, Figure 4a), while miR-221-3p only tended to be elevated and was not statistically different, likely due to the small sample size of samples from patients with IPMN associated with INV (Figure 4b). Overall, miR-10a-5p seemed to be a useful and biologically important biomarker.

## 3. Discussion

In this study, we showed that miR-10a and miR-221 were upregulated and that miR-148a was downregulated in IPMN associated with invasive carcinoma by NGS using tumor-normal paired resected tissues. In addition, we validated this finding by showing that miR-10a was upregulated in pancreatic juice samples from IPMN associated with invasive carcinomas, and it is a promising biomarker candidate for diagnosing malignant IPMN.

We identified miRNA biomarkers that were not previously reported in IPMN by performing an NGS analysis using tumor-normal paired tissues. A few reports [31,32,33,34] have detected differentially expressed miRNAs in IPMN, none of which reported elevated levels of miR-10a and miR-221 in malignant IPMN. Previous analyses of candidate miRNAs in IPMN reported that miR-21 and miR-155 were upregulated in IPMN [31] and invasive IPMN [32]. Comprehensive miRNA analyses revealed other miRNA biomarkers for malignant IPMN, such as miR-100, miR-99a, miR-99b, miR-342-3p, miR-126, miR-130a, miR-217, miR-216a, miR-216b, miR-148a, miR-375, miR-130b, miR-146b, miR-150, miR-214, miR-503, miR-21, miR-708 and miR-155 using microarrays [33,35], and miR-216a, miR-217, miR-802, miR-204, miR-218-1 and miR-214 using NGS [36]. Most of the obtained miRNAs demonstrated interstudy variations. One possible reason for the variation of results among studies was that internal controls for miRNA were not established, and another is that the background miRNA expression levels in each individual are likely to exhibit a strong effect on results. To overcome these problems, we performed a two-step normalization of the miRNA sequence data by, first, calculating the count data percentage for each miRNA per sample and then calculating the ratio of expression in the tumor and the adjacent acinus normal tissue. We believe that this two-step normalization enabled the validation of two miRNA biomarkers, miR-10a and miR-221, which were derived from tissue and pancreatic juice samples.

The newly identified biomarkers in IPMN, miR-10a and miR-221 are already known to be biologically meaningful miRNAs in other neoplasms. MiR-10a is upregulated in various tumors, including pancreatic cancer [37], especially in malignant and metastatic tumors [38,39,40], and exhibits functions, such as tumor migration and invasion through *PTEN* [41,42] and *HOXA1* [43]. This led us to presume that miR-10a was involved in the progression function of tumors as an oncomiR. MiR-221 also exhibits a progressive function in various tumors and interferes with tumor suppressor genes, such as *PTEN*, *CDKN1B* and *BLCL2L11* in pancreatic cancer cell lines [44,45,46]. These two miRNAs are also promising biomarkers; miR-10a may predict the advanced stage and prognosis in acute myeloid leukemia [47] and cervical cancer [48], and the development of pancreatic cancer [49]. miR-221 is also a candidate biomarker for pancreatic cancer [50]. These reports support the significance of our findings on miR-10a and miR-221 as clinically and biologically important biomarkers.

Multiple clinical implications of our study’s findings exist. First, new biomarkers for malignant IPMN should be validated in other cohorts and analyzed in subgroups depending on radiological morphologies, histological grades and epithelial types of IPMN, which could be added to the ICG in order to attain an improved risk stratification of IPMN. Second, our two-step normalization of the miRNA sequences should be validated in other studies. Although miRNAs are expected to be good biomarkers in various diseases because of their stable expression, the lack of definite internal controls remains a problem for their analysis. In this study, we provided a two-step normalization method to analyze miRNA sequence read data, which we believe will improve the universality of the obtained results. Third, due to their stable expression, even in poorly conditioned clinical samples that contain many enzymes, miRNAs are expected to be useful biomarkers in liquid biopsies. We showed that this was feasible in pancreatic juice samples, and we also expect miRNAs to be of clinical use in the future in samples such as plasma, duodenal juice and ascites.

This study has several limitations. First, the design was retrospective, and hence only a small number of cases were recruited from a single center. Therefore, we could not further analyze the associations between miRNA expressions, morphological classification and IPMN subtypes, although we recognize the importance of these analyses. Second, the miRNA analysis in pancreatic juice was performed only for miR-10a and miR-221 in order to avoid multiple statistical tests in a small sample.

In conclusion, this study provides new miRNA biomarkers for malignant IPMN via a miRNA sequence using tumor-normal paired tissues. After these biomarkers are validated by large cohorts with subgroups based on the radiological morphology, histological grades and epithelial type of IPMN, we hope that these biomarkers will provide additional benefits to the international consensus guideline for a better risk stratification of IPMN.

## 4. Materials and Methods

### 4.1. Patients and Samples

**Figure 5:**
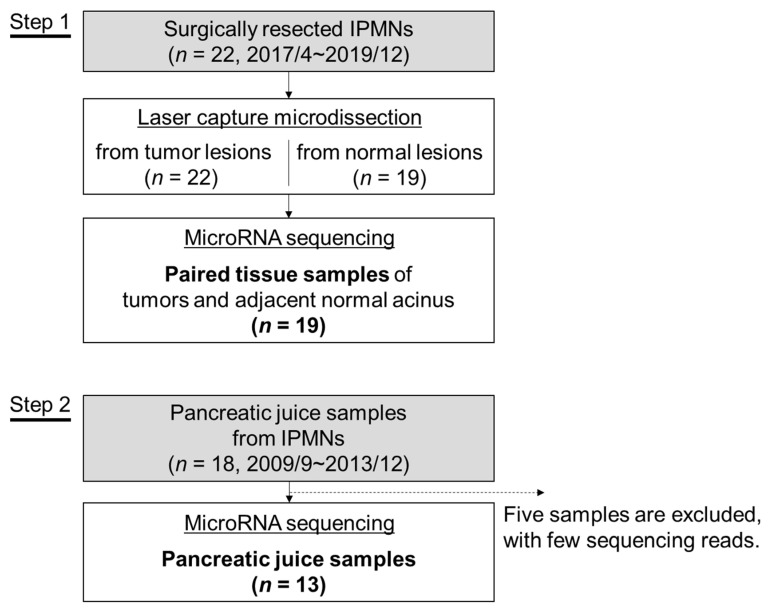
A flowchart of the study. The microRNA expression in intraductal papillary mucinous neoplasm (IPMN) was analyzed in tissue samples (Step 1) and pancreatic juice samples (Step 2).

We retrospectively analyzed resected tissues and pancreatic juice samples of 40 patients with IPMN who received surgical resections and/or systemic chemotherapy at Yamanashi University Hospital between September 2009 and November 2019. The study flow chart is shown in Figure 5, and the clinical characteristics of the enrolled patients are shown in Table 2 and Table S2. Tissues were obtained from resected specimens in which tumor components and their adjacent normal acini were separated by laser capture microdissection (LCM) using an ArcturusXT Laser Capture Microdissection System (Life Technologies, Carlsbad, CA, USA) from 8-μm thick sections of FFPE samples. Representative histological images of LGD, HGD and invasive IPMN (INV) are shown in Figure S1. Samples were excluded if a paired normal acinus sample was not available. The tumor tissues and their paired normal acini were subjected to comprehensive miRNA analysis by NGS (*n* = 19). According to the manufacturer’s specifications, the total RNA including miRNA was extracted from LCM specimens with the AllPrep DNA/RNA FFPE Kit (QIAGEN, Hilden, Germany). Pancreatic juice samples were collected via preoperative endoscopic retrograde pancreatography from 18 patients who underwent surgical resection between 2009 and 2013. The patients whose tissues were analyzed were an independent cohort from those whose pancreatic juice samples were analyzed. MiRNAs were extracted using the miRNeasy Mini Kit (QIAGEN, Hilden, Germany) from 500 µL of pancreatic juice samples. Of the 18 pancreatic juice samples, five were excluded from the analysis because of few sequence reads in the downstream NGS analysis (Figure 5). The quantity and quality of the extracted RNA was assessed by a NanoDrop (Thermo Fisher, Waltham, MA, USA) instrument with the Qubit platform (Thermo Fisher, Waltham, MA, USA), and the small RNA fraction including miRNA was assessed by an RNA 6000 Nano Kit on the Agilent 2100 Bioanalyzer on-chip electrophoresis (Agilent Technologies, Santa Clara, CA, USA). This study was approved by the Human Ethics Review Committee of Yamanashi University Hospital (Receipt numbers: 1326 and 1847), and written informed consent was obtained from all patients.

### 4.2. RNA Sequencing for miRNA Expression Analysis

The extracted total RNA or miRNA was enriched for small RNA using a Magnetic Bead Cleanup Module (Thermo Fisher, Waltham, MA, USA), as per the manufacturer’s instruction. The quality and quantity of the enriched small RNAs were assessed by the Agilent 2100 Bioanalyzer instrument with the Agilent Small RNA Kit (Agilent Technologies, Santa Clara, CA, USA). Then, the small RNAs were reverse-transcribed using the Invitrogen™ SuperScript™ VILO™ cDNA Synthesis Kit (Thermo Fisher, Waltham, MA, USA). Small RNA libraries for sequencing were generated using the Ion Total RNA-Seq Kit v2 (Thermo Fisher, Waltham, MA, USA) with the Ion Xpress Barcode Adapters Kit (Thermo Fisher, Waltham, MA, USA) for multiplexing samples. Barcoded libraries were amplified using an emulsion polymerase chain reaction on Ion Sphere particles, and sequencing was performed on an Ion Chef System and an Ion Proton Sequencer (Life Technologies, Carlsbad, CA, USA) using an Ion PI Hi-Q Chef Kit (Life Technologies, Carlsbad, CA, USA). Sequenced reads were trimmed, their quality assessed by a program called FastQC, aligned to the reference genome (hg19) and then counted for miRNA using the SmallRNA Analysis program on the Torrent Suite Software (Thermo Fisher, Waltham, MA, USA).

### 4.3. Analysis of Count Data for Differentially Expressed miRNAs

The count data for each miRNA were normalized by calculating the percentage of each miRNA count in the total sample as follows: “Normalized miRNA expression = (Read count of each miRNA)/(All read counts in the sample).” The tumor-to-normal expression ratio was calculated as follows: “Normalized miRNA expression of the tumor tissue samples/Normalized miRNA expression of the adjacent normal acinus tissue samples.” The count data in pancreatic juice samples were normalized and analyzed without a normal denominator.

A heatmap of miRNA expressions with pathological grades of IPMN was created by showing the miRNA expression levels on a green-red scale in a panel where the green and red scales indicated a low and high expression, respectively. Patients and miRNAs were arranged according to an unsupervised hierarchical clustering, which was performed using a one minus Pearson’s correlation as the distance measure and an average linkage as the agglomerative method.

### 4.4. Statistical Analysis

Comparisons of the miRNA expression within groups and between tumor tissues and their adjacent normal acinus were evaluated using the Kruskal–Wallis test and Mann–Whitney U test, respectively, and *p* < 0.05 was considered significant. All statistical analyses were performed using the statistical functions built into the Excel software (Microsoft Corporation, Redmond, WA, USA), and a volcano plot analysis was made by plotting the significance versus fold-change on the y- and *x*-axis, respectively.

## Figures and Tables

**Table 1:** A post hoc analysis of 15 microRNAs that exhibited significantly different expression patterns among three histological grades (Kruskal–Wallis test, *p* < 0.05).

	LGD vs. HGD	LGD vs. INV	HGD vs. INV
	*p* Value	*p* Value	*p* Value
miR-210-3p	0.949	0.133	0.026 *
miR-221-3p †	0.811	0.049 *	0.048 *
let-7i-5p	0.271	0.028 *	0.276
miR-10a-5p †	1	0.049 *	0.009 *
miR-598-3p	0.078	0.028 *	0.333
miR-200a-3p	0.624	0.203	0.036 *
miR-200b-3p	0.889	0.203	0.036 *
miR-200c-3p	0.889	0.083	0.036 *
miR-660-5p	0.347	0.407	0.036 *
miR-362-3p	0.206	0.133	0.026 *
miR-29c-3p	0.811	0.083	0.026 *
miR-340-5p	0.949	0.083	0.009 *
miR-148a-3p †	0.271	0.028 *	0.004 *
let-7b-5p	0.526	0.133	0.048 *
miR-193b-3p	0.624	0.133	0.036 *

LGD, low-grade dysplasia; HGD, high-grade dysplasia; INV, invasive IPMN; *, *p* < 0.05; †, Statistically significant in both LGD vs. INV and HGD vs. INV.

**Table 2:** Summary of clinicopathological features.

	Tissue	Pancreatic Juice
	(*n* = 19)	(*n* = 13)
Age, median (range), years	74 (54–84)	74 (61–79)
Sex, *n* (%)		
Male	12 (63)	5 (38)
Female	7 (37)	8 (62)
Morphological classification, *n* (%)		
Branch-duct type	10 (53)	5 (38)
Main-duct type	4 (21)	4 (31)
Mixed type	5 (26)	4 (31)
Grade of IPMN, *n* (%)		
Low-grade dysplasia	4 (21)	8 (62)
High-grade dysplasia	9 (47)	2 (15)
Invasive carcinoma	6 (32)	3 (23)
UICC stage of invasive carcinoma, *n*		
IIA/IIB	1/5	2/1
Epithelial type of IPMN, *n* (%)		
Gastric	6 (32)	9 (69)
Intestinal	2 (11)	3 (23)
Pancreatobiliary	1 (5)	1 (8)
Oncocytic	1 (5)	0
Unknown	9 (47)	0

## Data Availability

Research data obtained in this study are not shared.

## Supplementary Materials

The following are available online at https://www.mdpi.com/1422-0067/22/6/3221/s1. Table S1: Summary of aligned reads of RNA sequences for miRNA expressions from tissue and pancreatic juice samples; Table S2: Detailed patients’ characteristics; Figure S1: Representative pathological images of LGD, HGD and invasive IPMN (INV).
