# Supplementary material for: MiR-10a in Pancreatic Juice as a Biomarker for Invasive Intraductal Papillary Mucinous Neoplasm by miRNA Sequencing
Source: Int J Mol Sci. 2021 Mar 22;22(6):3221. doi: 10.3390/ijms22063221 (PMC8004614; doi:10.3390/ijms22063221)
Supplement: Supplementary file 1 [file ijms-22-03221-s001.pdf]

Supporting information

Table S1. Summary of aligned reads of RNA sequence for miRNA expressions from tissues and pancreatic juice samples

Small RNA-seq	Tissue (n = 38)	Pancreatic juice (n = 13)
Aligned reads	253,659 (35,795–1,140,698)	117,798 (11,700–1,077,040)
miRNA reads	36,485 (3,147–200,540)	34,168 (1,582–344,562)
Reference miRNA	2,792	2,792
Detected miRNA	176 (57–319)	94 (15–326)
Analyzed miRNA	194	162

Table S2. Detailed patients' characteristics.

Tissue (n = 19)				Pancreatic juice (n = 13)			
Case	Grade of IPMN	Morphological classification	Epithelial type	Case	Grade of IPMN	Morphological classification	Epithelial type
T1	HGD	BD	N/A	PJ1	HGD	MD	I
T2	INV	Mixed	N/A	PJ2	LGD	BD	G
T3	LGD	MD	G	PJ3	INV	MD	I
T4	HGD	Mixed	N/A	PJ4	LGD	BD	G
T6	INV	BD	PB	PJ5	LGD	BD	G
T8	LGD	BD	N/A	PJ7	INV	Mixed	PB
T9	HGD	Mixed	N/A	PJ9	LGD	Mixed	G
T10	HGD	BD	N/A	PJ10	INV	Mixed	G
T11	INV	BD	I	PJ11	LGD	BD	G
T13	LGD	BD	G	PJ13	LGD	Mixed	I
T14	HGD	MD	I	PJ15	LGD	BD	G
T16	HGD	BD	Onco	PJ16	HGD	MD	G
T17	INV	MD	G	PJ18	LGD	MD	G
T18	LGD	Mixed	G				
T19	HGD	BD	N/A				
T20	HGD	BD	G				
T21	HGD	MD	G				
T22	INV	BD	N/A				
T23	INV	Mixed	N/A				

LGD, low-grade dysplasia; HGD, high-grade dysplasia; INV, IPMN with an associated invasive carcinoma;
BD, branch-duct type; MD, main-duct type; N/A, not available

G, gastric type; I, intestinal type; PB, pancreatobiliary type; Onco, oncocytic type;

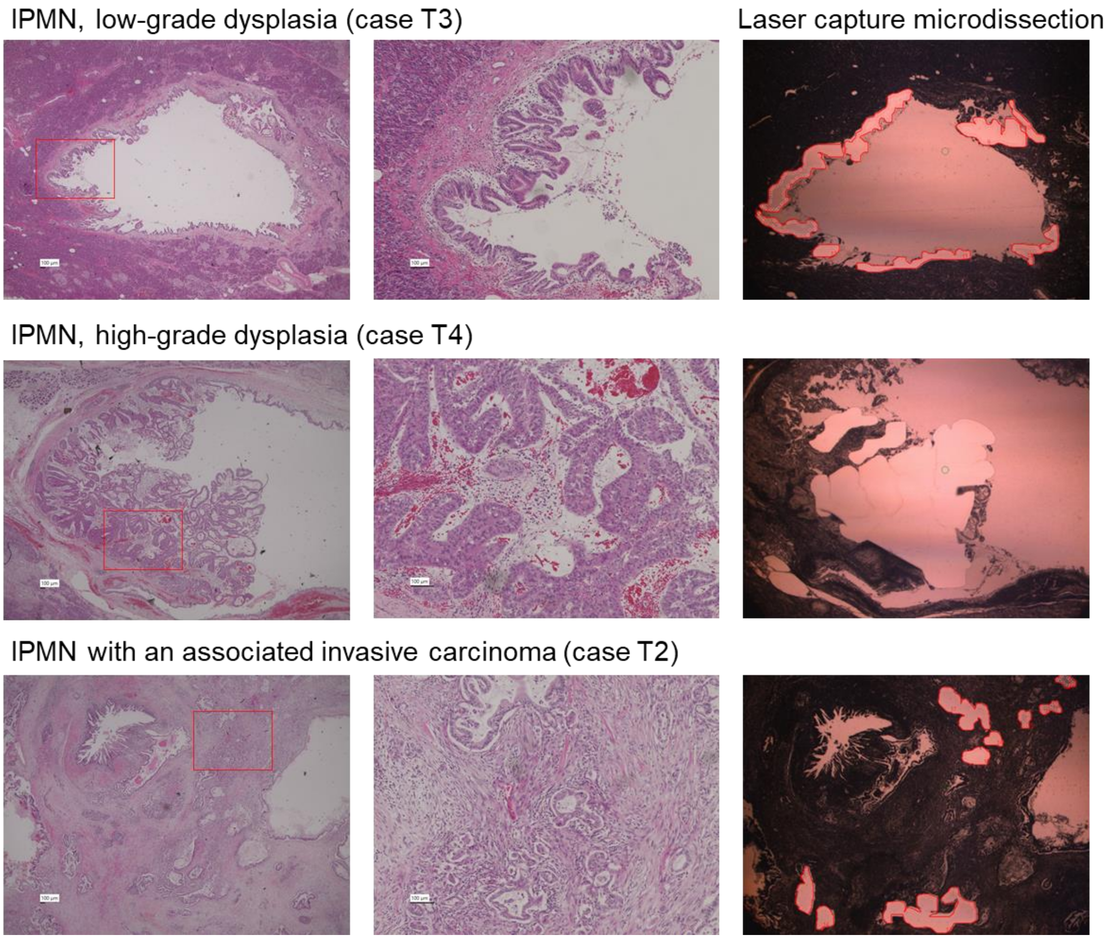

Figure S1. Representative pathological images of LGD, HGD, and invasive IPMN (INV).
